# Intraoperative Near-Infrared Autofluorescence and Indocyanine Green Imaging to Identify Parathyroid Glands: A Comparison

**DOI:** 10.1155/2019/4687951

**Published:** 2019-09-25

**Authors:** Max Lerchenberger, Norah Al Arabi, Julia K. S. Gallwas, Herbert Stepp, Klaus K. J. Hallfeldt, Roland Ladurner

**Affiliations:** ^1^Department of Surgery, Ludwig Maximilians University Munich, Innenstadt Medical Campus, Nussbaumstrasse 20, 80336 Munich, Germany; ^2^Department of Obstetrics and Gynecology, Ludwig Maximilians University Munich, Maistr. 11, 80337 Munich, Germany; ^3^Laser-Research Laboratory, LIFE-Center and Department of Urology, Ludwig Maximilians University Munich, Grosshadern Medical Campus, Feodor-Lynen-Str. 19, 81377 Munich, Germany

## Abstract

**Objective:**

To investigate the feasibility of near-infrared autofluorescence (AF) and indocyanine green (ICG) fluorescence to identify parathyroid glands intraoperatively.

**Methods:**

Fluorescence imaging was carried out during open parathyroid and thyroid surgery. After visual identification, parathyroid glands were exposed to near-infrared (NIR) light with a wavelength between 690 and 770 nm. The camera of the Storz® NIR/ICG endoscopic system used detects NIR light as a blue signal. Therefore, parathyroid AF was expected to be displayed in the blue color channel in contrast to the surrounding tissue. Following AF imaging, a bolus of 5 mg ICG was applied intravenously. ICG fluorescence was detected using the same NIR/ICG imaging system. Well-vascularized parathyroid glands were expected to show a strong fluorescence in contrast to surrounding lymphatic and adipose tissue.

**Results:**

We investigated 78 parathyroid glands from 50 patients. 64 parathyroid glands (82%) displayed AF showing the typical bluish violet color. 63 parathyroid glands (81%) showed a strong and persistent fluorescence after application of ICG. The sensitivity of identifying a parathyroid gland by AF was 82% (64 true positive and 14 false negative results), while ICG imaging showed a sensitivity of 81% (63 true positive and 15 false negative results). The Fisher exact test revealed no significant difference between both groups at *p* < 0.05. Neither lymph nodes nor adipose tissue revealed substantial AF or ICG fluorescence.

**Conclusion:**

AF and ICG fluorescence reveal a high degree of sensitivity in identifying parathyroid glands. Further, ICG imaging facilitates the assessment of parathyroid perfusion. However, in the current setting both techniques are not suitable as screening tools to identify parathyroid glands at an early stage of the operation.

## 1. Introduction

Difficulties in accurately localizing parathyroid glands during thyroid surgery may result in the disruption of the parathyroid vasculature. Despite refined operation techniques, postoperative temporary hypocalcemia remains the most common complication after total thyroidectomy [1–4]. The standard procedure to identify and save parathyroid glands is just by visual inspection, and the result largely depends on the surgeon's experience. Most endocrine surgeons would agree that there is a great demand for a simple and reliable technique to identify parathyroid glands intraoperatively. To date, numerous localization techniques have been proposed to facilitate intraoperative parathyroid identification.

The idea of using a dye or a fluorophore in order to visualize parathyroid glands is not new. Back in 1971, Dudley described the application of methylene blue as an exogenous contrast agent [5]. 30 years later, van der Vorst et al. investigated near-infrared fluorescence imaging based on the intravenous application of small doses of methylene blue [6], and 20 years ago, Prosst et al. proposed aminolevulinic acid (ALA) as a contrast agent [7]. Although these methods proved to be effective in trials, they could not become generally accepted, and the clinical approach to parathyroid identification remained stagnant [8–10]. Rubinstein et al. have been the first to visualize parathyroid glands by applying optical coherence tomography (OCT), a noninvasive high-resolution imaging technique that permits characterization of microarchitectural features up to 2 mm in depth [11]. OCT images of lymph nodes and parathyroid, thyroid, and adipose tissue display typical characteristics for each entity, facilitating a reliable differentiation. Conti de Freitas et al. and our research group were able to confirm these favorable results ex vivo [12–14]. However, due to technical problems, handling the OCT probe covered with a sterile sheath it was impossible to obtain similar results in vivo [15].

The recent discovery of parathyroid autofluorescence by a research team of biomedical engineers and endocrine surgeons from Nashville, Tennessee has renewed the interest in intraoperative parathyroid imaging [16]. At an excitation wavelength in the near-infrared (NIR), parathyroid tissue exhibits a unique autofluorescence up to 11 times higher than that of the surrounding tissue. As most tissue types are more or less completely void of autofluorescence in this wavelength range, even a weak fluorescence signal can provide a high contrast. Furthermore, this wavelength range is characterized by a high penetration depth and allows for the recognition of fluorescent tissue. In the meantime, a large number of studies have confirmed the excellent detection rates initially described by McWade et al. [17–26].

In 2016, Zaidi et al. described the use of indocyanine green (ICG) fluorescence imaging during parathyroid and thyroid surgery [27, 28]. In these prospective studies, over 90% of parathyroid glands were reliably identified by their ICG uptake. In the same year, Fortuny et al. reported on a study using ICG angiography in order to assess parathyroid vascularization [29]. By now, the high sensitivity in detecting parathyroid glands using ICG imaging and just as well screening their vascularization has been supported by numerous studies [30–32].

Autofluorescence and ICG fluorescence imaging represent the techniques most intensively investigated at present. In this in vivo study we compare the different approaches and try to assess their differences.

## 2. Materials and Methods

This prospective in vivo study was approved by the institutional ethical review board of the Medical faculty of the University of Munich. Patients undergoing open hemithyroidectomy or total thyroidectomy and patients undergoing open parathyroidectomy with complete cervical exploration were eligible for enrollment. Informed consent was obtained from all patients.

### 2.1. Imaging System

Parathyroid ICG fluorescence was visualized using a commercially available near-infrared/indocyanine green (NIR/ICG) endoscopic system (Karl Storz, Tuttlingen, Germany). The system comprises a high-end full high-definition camera system (Image1 H3-Z 3-Chip Full HD camera, Karl Storz) connected to a 10 mm 0° ICG telescope (Hopkins™ II, Karl Storz). The camera is sensitive for NIR light with its blue channel, due to the spectral properties of the dielectric coatings of the color beamsplitter. The telescope is equipped with a specific filter for optimal detection of white light and NIR fluorescence, while completely blocking out NIR excitation light. In the NIR excitation mode, the light source also emits low-intensity light in the green and red spectral ranges to enable orientation during NIR fluorescence imaging. The system's xenon light source (D-Light P, Karl Storz) provides both visible and NIR excitation light at a wavelength of 690 nm to 790 nm. The surgeon can use a footswitch to change from white light to NIR. Parathyroid autofluorescence was detected using the same NIR/ICG endoscopic system as its excitation wavelength in the near-infrared is around 800 nm, and autofluorescence is emitted at around 820 nm. However, as autofluorescence is considerably weaker than ICG fluorescence and previous investigations showed a notable amount of light being backscattered and recorded in the blue channel, a second xenon light source (D-Light P, Karl Storz) was modified by interposing an additional longpass filter. Further, a bandpass filter was added to reduce the light in the green and red spectral region [20].

### 2.2. Intraoperative Imaging

Autofluorescence and ICG imaging were carried out during open thyroid and parathyroid surgery for benign and malignant disease. After lateral mobilization of the thyroid and exposure of the recurrent laryngeal nerve, the parathyroid glands were visualized. However, we did not search for a gland when it was not apparent during initial thyroid dissection. Using magnifying glasses special care was taken to preserve the vascular pedicles. Only parathyroid glands that were definitely identified by an experienced endocrine surgeon were considered for fluorescence imaging. First, white light images were collected. Second, with all operating room lights turned off, the parathyroid gland as well as the surrounding tissue were exposed to near-infrared (NIR) light. The tip of the laparoscope was held stationary approximately 5 cm above the tissue. With the light source in fluorescence mode, NIR light was detected as a blue signal by the camera, and in contrast to the surrounding tissue the parathyroid gland was expected to be displayed in the blue color channel. The autofluorescent effect was photo-documented for each parathyroid gland.

For ICG fluorescence imaging, 25 mg of the fluorophore ICG-Pulsion® (Diagnostic Green GmbH, Aschheim-Dornach, Germany) was dissolved in 5 ml sterile water and 5 mg (1 ml) injected intravenously. ICG fluorescence became apparent after 1-2 minutes. The progression was video-documented over 3–5 minutes. To allow a direct comparison between autofluorescence and ICG fluorescence, we investigated only one side in each patient. Measuring autofluorescence after application of ICG on the contralateral side would have falsified the results for AF imaging.

When a parathyroid gland revealed at least the same fluorescence than the surrounding thyroid tissue, ICG fluorescence was regarded as being positive. In most cases, the fluorescence even persisted after the decrease of thyroid fluorescence. A negative result was defined as parathyroid fluorescence being completely missing or being significantly less compared to the surrounding thyroid tissue.

## 3. Results

Between October 2017 and May 2018, 78 parathyroid glands from 50 patients who underwent open thyroid or parathyroid surgery were examined with regard to their autofluorescence and ICG fluorescence. The demographic and clinical details are shown in Table 1.

**Figure 1:**
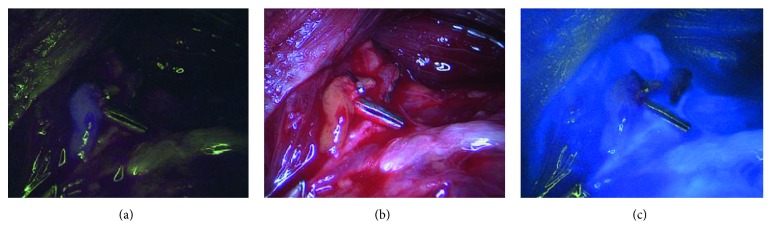
Parathyroid gland. (a) Autofluorescence image that displays the typical bluish violet color of the gland. (b) The parathyroid gland in normal white light. (c) 2 min after i.v. application of 5 mg ICG. The gland shows hardly any ICG fluorescence, and it can be assumed that it is devascularized.

**Figure 2:**
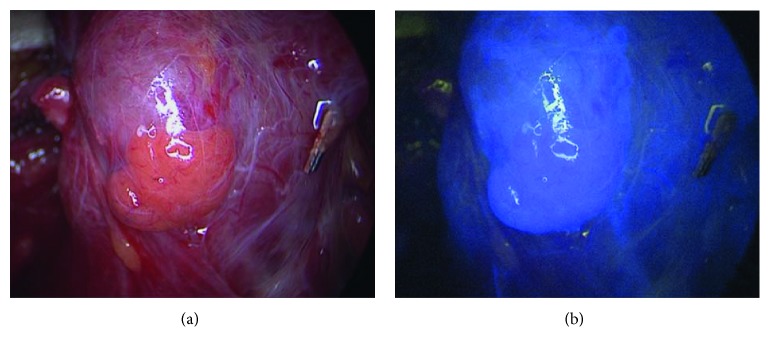
Parathyroid gland exposed to normal white light (a) and near-infrared light 2 min after i.v. application of 5 mg ICG (b). The gland displays a strong fluorescence indicating a good vascularity. The surrounding structures, especially the thyroid, are less fluorescent.

64 parathyroid glands (82%) displayed NIR autofluorescence showing the typical bluish violet color as described in previous studies [20–22]. The intensity of their fluorescence enabled a sharp distinction from surrounding structures, especially lymph nodes, thyroid tissue, and adipose tissue (Figure 1(a)). Regarding the extent of autofluorescence, we could not observe noticeable differences between vascular and avascular glands. Autofluorescence imaging was especially helpful in identifying inferior parathyroid glands during central lymph node dissection. In 14 cases (18%) we were unable to visualize autofluorescence despite unambiguous visual identification of the parathyroid gland (Table 2). The sensitivity of identifying a parathyroid gland by autofluorescence was 82%. ICG imaging was carried out subsequent to autofluorescence imaging. 63 parathyroid glands (81%) showed a persistent fluorescence after the decrease of thyroid fluorescence. This effect was seen 2-3 min after i.v. ICG application (Figure 2). In 11 cases (14%) fluorescence was not observed, although visual inspection suggested a good vascularity of the parathyroid tissue, and no change of color was noted during the operation (Figures 1(b) and 1(c)). 4 parathyroid glands showed a distinct darkening of color following dissection.

The subsequent ICG application did not induce a noticeable fluorescence, and we had to assume a damage to parathyroid vascularization. These glands were autotransplanted into the sternocleidomastoid muscle. Two of these patients developed transient hypocalcemia postoperatively which was treated with calcium effervescent tablets 4 × 500 mg/die and alfacalcidol capsules 1 *µ*g/die. Further, all patients with thyroidectomy and central lymph node dissection received this medication on a routine basis as well as 3 patients with Graves' disease and moderate ICG fluorescence, where we had to dissect the inferior parathyroid glands from the thyroid capsule.

The sensitivity of identifying a parathyroid gland by autofluorescence was 82% (64 true positive and 14 false negative results) while ICG imaging showed a sensitivity of 81% (63 true positive and 15 false negative results). The Fisher exact test revealed no significant difference between both groups at *p* < 0.05. However, the quoted sensitivity of ICG imaging must be judged with caution as the technique relies on well-vascularized tissue, and a compromised circulation falsifies the results.

Regarding the extra time necessary to perform AF imaging, we required approximately 3–5 minutes to prepare the imaging system and 2-3 min to visualize both parathyroid glands on one side. ICG imaging was more time-consuming with around 3 extra minutes to see the peak of parathyroid fluorescence emission.

## 4. Discussion

The study demonstrates that both imaging techniques examined are helpful in identifying parathyroid glands intraoperatively. Autofluorescence and ICG imaging showed a sensitivity of 82% and 85%, respectively. There was no statistically significant difference between both groups. These results are consistent with observations made by other authors who reported detection rates of 77–100% with AF [19–22, 25] and 84–100% with ICG fluorescence [29–31, 33, 34].

However, both techniques are currently not sufficiently sensitive to operate as screening tools helping to localize parathyroid tissue at an early stage of the operation. The initial goal, the precise and rapid detection of parathyroid glands by way of screening the surgical site, cannot be achieved. Frequently, parathyroid glands are covered by a sheath of adipose tissue which needs to be removed in order to display autofluorescence. We experienced similar problems with ICG imaging, where adipose tissue but also even minor bleeding at the operating site may obscure the fluorescence. AF and ICG imaging may be helpful to confirm the presence of a parathyroid gland, but in their present utilization the techniques will not be effective to replace accurate dissection by an experienced surgeon. Regarding AF, more sensitive imaging systems than those available at present could lead to an effective screening at an early stage of the operation and replace the present strategy of confirming the presence of a visually identified parathyroid gland. For ICG imaging we used a small dose of 5 mg indocyanine per patient. In our opinion, it would be of interest to investigate whether higher doses increase parathyroid fluorescence and thereby allow an early detection. The cause for the longer persistence of ICG-fluorescence in parathyroid glands as compared to the thyroid is not known to us. A possible explanation may be a slower venous blood flow.

Table 3 summarizes advantages and disadvantages of autofluorescence and ICG imaging as experienced in our study. All investigations were carried out using a commercially available Storz ICG imaging system (Karl Storz GmbH, Tuttlingen, Germany) with only slight modifications to the light source for autofluorescence imaging. Irrespective of their ICG components, camera and light source provide the same features as the standard laparoscopic equipment and can be used for routine laparoscopic surgery. The additional costs for the NIR imaging capability amount to approximately 4000 Euro. A 25 mg vial of indocyanine green (ICG-Pulsion®, Diagnostic Green GmbH) costs around 70 Euro.

The commercially available Fluobeam® 800 system (Fluoptics, Grenoble, France) represents another, meanwhile well-established, device to detect parathyroid autofluorescence. A laser provides an irradiance of 5 mW/cm^2^ at 750 nm and collects the optical signal for wavelengths above 800 nm [24–26]. At an experimental level, Kim et al. introduced a new technique to visualize both parathyroid glands and the surrounding tissue in a single image, using a 780 nm collimated light-emitting diode (Thorlabs, Newton, NJ, USA) with an excitation filter appropriate for parathyroid autofluorescence, an illuminator (INFRALUX-300, Daekyoung, Korea) for reflection of the entire surgical area, and a digital single-lens reflex camera (Canon, EOS REBEL T3, Ota, Tokyo, Japan) [23, 27].

Other commercially available devices used for ICG fluorescence imaging were the Novadaq® Pinpoint system (Novadaq®, Mississauga, On, Canada) and the Striker® endoscopic near-infrared visualization(ENV) system (Stryker® Endoscopy, San Jose, CA, USA). The main difference of these devices compared to the Storz ICG imaging system is the use of a NIR laser instead of a xenon light source. To our knowledge, no studies exist that directly compare their performance.

Along with higher costs, longer operating times may be an obstacle to incorporate a new technique to conventional surgery. In our experience, the additional time needed for AF imaging and ICG imaging amounts to 5–10 min and 8–13 min, respectively. This includes setting up the endoscopic system, darkening the operating room, and endoscopically localizing the parathyroid glands. With extended i.v. lines, as in our setting due to the positioning of the arms, an extra 3 min was needed to follow ICG enhancement in the tissue with the peak to be seen in the parathyroid glands after 2-3 min. AF reveals no difference between vascularized and devascularized glands. In contrast, ICG imaging depends on tissue vascularity and as described by several authors it is well possible to identify devascularized glands (Figure 2) and thus, simplifying the decision for autotransplantation. In the present series, 15 out of 78 parathyroid glands did not reveal an adequate ICG fluorescence, most likely caused by insufficient vascularization. As we did not notice a change of color in 11 of these glands, during the further course of the operation we refrained from autotransplantation. We believe, the disruption may have been temporarily, for instance by vascular compression or traction on the thyroid lobe. A complete separation of the parathyroid vessels would have been very unlikely at the early stage of the operation when ICG imaging was carried out.

Apart from assessing parathyroid vascularization intraoperatively, ICG imaging may be helpful to visualize the exact vascular anatomy before dissection close to the gland begins. This could be a major advantage in order to avoid hypoparathyroidism, especially if parathyroid glands are attached to the thyroid or possess a long vascular pedicle [35].

A limitation of the present study is that we had to rely on the visual evaluation of parathyroid glands. Although we carried out measurements only in situations where we were absolutely sure to have identified parathyroid tissue, 11 of the 15 ICG negative results and especially the 7 ICG and AF negative results must be questioned. As already discussed in previous studies [20–22], we cannot explain why in our series AF imaging failed in around 15% of cases. The cause could be our imaging system, originally destined to provide ICG imaging but not to display the considerably weaker parathyroid autofluorescence. Further, in a recent article McWade et al. noted that different probe placements could lead to different measurements from the same gland [19]. These differences were attributed to intraglandular heterogeneity. In our experience, AF becomes more difficult to detect when the glands are embedded in adipose tissue or seem to contain a high amount of adipose cells. Regarding parathyroid vascularity, we noticed substantial differences between our visual impression and ICG imaging. 11 parathyroid glands showed no darkening in color despite a missing or poor contrasting after application of indocyanine green. This suggests that ICG imaging may be more precise to evaluate parathyroid vascularity and that a missing change of color does not imply good vascularization.

In practical terms, we found it difficult to apply the ICG 0–2 scoring system proposed by Fortuny et al. [30] and especially to distinguish between grey, grey/black, and entirely black parathyroid glands. This may be due to a lack of experience and certainly this new technique involves a learning curve. In this study, the indication for autotransplantation still depended on the visual impression of the gland, and it was not our intention to correlate ICG fluorescence and postoperative hypocalcemia.

As mentioned before, both techniques are not screening tools, and parathyroid glands need to be identified beforehand with the naked eye. AF imaging allows to localize parathyroid glands even though the tissue is devascularized. We found this helpful in surgery with central lymph node dissection, either to verify their localization or to confirm their identity before autotransplantation. For ICG imaging, parathyroid glands need to be vascularized. This circumstance may be used to predict postoperative parathyroid function [30, 31, 33, 34] or in future to assist the decision making whether an autotransplantation should be carried out.

## 5. Conclusions

Although most parathyroid and thyroid operations are uncomplicated, difficult situations occur regularly, and it may be crucial to identify and preserve parathyroid tissue. It seems to be premature to assume that AF imaging or ICG imaging will play a major role regarding the intraoperative identification of parathyroid glands. However, both techniques have so far shown to be fairly reliable. They can be easily applied intraoperatively and require only moderate expenditure. However, the main issue, the precise and rapid detection of parathyroid glands by way of screening the surgical site, cannot be achieved.

## Figures and Tables

**Table 1:** Descriptive data.

Number of patients	50
Mean age	47.2 years
Sex (female/male)	36/14
Type of operation	(*n*)
Open parathyroidectomies	17
Thyroidectomies—Goiter	7
Thyroidectomies—Graves' disease	9
Thyroidectomies—Carcinoma	8
Plus central LN dissection	4
Hemithyroidectomies	5

**Table 2:** Comparison of autofluorescence (AF) and indocyanine green (ICG) fluorescence.

	AF positive	AF negative	Total
ICG positive	56	7	63
ICG negative	8 (4 devas.)	7	15
Total	64	14	78

78 parathyroid glands were examined. 64 showed autofluorescence (sensitivity 82%) and 63 ICG fluorescence (sensitivity 85%). 7 glands were negative both for AF and ICG fluorescence. 4 ICG negative glands showed a darkening of color intraoperatively and we had to assume their devascularization. The Fisher exact test did not reveal significant differences between both the groups.

**Table 3:** Comparison of autofluorescence and ICG imaging of parathyroid glands.

Autofluorescence imaging	ICG imaging
Confirmation of parathyroid tissue after identification of the gland by the naked eye	Confirmation of parathyroid tissue after identification of the gland by the naked eye

No screening tool	No screening tool

No administration of a pharmaceutical	Intravenous administration of a fluorophore with potential side effects. Not approved for this indication in Europe.

Fluorescence imaging with a modified ICG system	Fluorescence imaging with a standard ICG system

No assessment of parathyroid perfusion	Assessment of parathyroid perfusion possible

No appraisal of vascular anatomy	Evaluation of vascular anatomy possible

Additional operating time: 5–10 min	Additional operating time: 8–13 min

No additional costs	Additional costs of 70 EUR (ICG)

## Data Availability

The data used to support the findings of this study are included within the article.
